# Use of Emergency Manuals to Treat Intraoperative Supraventricular Tachycardia and Hypotension During Exploratory Laparotomy

**DOI:** 10.7759/cureus.8828

**Published:** 2020-06-25

**Authors:** Kyle Sanchez, Daniel Eskander, Islaam Elnagar, Jeffrey Huang

**Affiliations:** 1 Miscellaneous, University of Central Florida College of Medicine, Orlando, USA; 2 Anesthesiology, HCA Healthcare/University of South Florida Morsani College of Medicine, Tampa, USA; 3 Anesthesiology, HCA Healthcare/University of South Florida Morsani College of Medicine GME/Oak Hill Hospital, Brooksville, USA; 4 Anesthesiology, University of Central Florida College of Medicine, Orlando, USA

**Keywords:** anesthesiology, emergency manuals, intraoperative tachycardia, intraoperative arrhythmia, emergency manual use

## Abstract

Although intraoperative tachyarrhythmias are relatively common, their appropriate management is pertinent to reducing morbidity and mortality. In certain clinical scenarios, the initial steps of managing intraoperative tachyarrhythmias may be ambiguous. Emergency manuals (EMs) are cognitive aids that improve the outcome of critical events by providing current, medically established guidelines on management. The case of a patient with an intraoperative supraventricular tachycardia with narrow, irregular QRS complexes and refractory hypotension is described here. Relevant sections of Stanford Anesthesia Emergency Manual were activated immediately and guided the anesthesiologists in treating the patient’s arrhythmia. The utilization of an EM allowed rapid selection of a pharmacologic agent that achieved hemodynamic stability. EMs allow healthcare providers to respond more appropriately and efficiently during critical events and thus directly improve patient care.

## Introduction

Intraoperative tachyarrhythmias are common and affect an estimated one million elderly American patients annually [1]. They are associated with significant morbidity and mortality, especially in elderly individuals with multiple co-morbidities [1-4]. In susceptible patients with pre-existing cardiovascular disease, even transient episodes of tachycardia can lead to significant myocardial ischemia secondary to reduced diastolic filling time. Intraoperative tachyarrhythmias, thus, must be corrected quickly and appropriately to reduce the risk of potentially fatal cardiac events.

However, medical errors in the intraoperative setting are common, and up to 11% of errors involving critically ill patients may be life-threatening [5]. Cognitive aids have been shown to positively influence the outcome of critical events by increasing immediate access to resources, and decreasing reliance on rote memory, which helps to reduce medical errors [6-7]. Operating room emergency manuals (EMs) are cognitive aids that contain current, medically established guidelines on the management of specific perioperative critical events [8]. The use of operating room EMs has been shown to reduce medical error and maximize productivity by allowing healthcare providers, especially anesthesia professionals, to respond more confidently and collaboratively to critical events [9-11].

## Case presentation

An 89-year-old male presented to the ED with abdominal pain, nausea, vomiting, and constipation of four days duration. His past medical history included hypertension, hyperlipidemia, type 2 diabetes mellitus, stage 3 chronic kidney disease, atrial fibrillation, and coronary artery disease with a prior coronary artery bypass graft. On exam, he was hypotensive and tachycardic with diffuse rebound tenderness and muscle guarding. Cardiopulmonary exam was otherwise benign. The patient was admitted to the hospital and received conservative treatment for bowel obstruction. His atrial fibrillation was treated with diltiazem 10 mg and metoprolol 5 mg. However, conservative management did not relieve his bowel obstruction, and exploratory laparotomy was scheduled. His preoperative cardiac work-up revealed an ejection fraction of 35%, and his pre-operative labs were significant for a hemoglobin of 9.7 g/dL (13.5-17.5 g/dL), but otherwise within normal limits. The patient was categorized as American Society of Anesthesiology Class IV.

**Figure 1:**
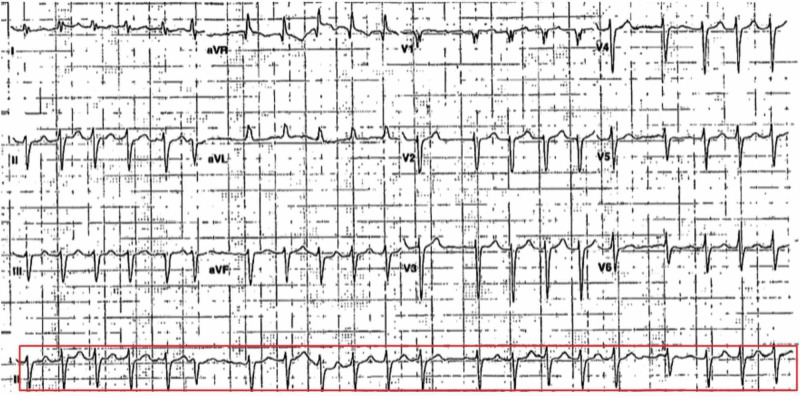
Intraoperative ECG showing atrial fibrillation with rapid ventricular response. ECG, electrocardiogram

The patient’s preoperative blood pressure was 130/75 mmHg, and his atrial fibrillation was under control with a heart rate of 89 beats per minute. The patient was brought to the operating room with standard monitors applied, and anesthesia was induced in the supine position with etomidate, fentanyl, and rocuronium. After intubation, anesthesia was maintained with sevoflurane in 100% oxygen. Left radial artery was cannulated with 20G cannula. The patient remained hemodynamically stable until three hours post-induction, when he became hypotensive at 65/40 mmHg. Intravenous (IV) normal saline and intermittent phenylephrine were administered. Subsequently, a central venous catheter was placed in the right internal jugular vein under ultrasonic guidance for additional IV access and central venous pressure (CVP) monitoring. After 5,000 mL of IV isotonic fluid, his CVP was elevated at 10 mmHg (2-8 mmHg) and his blood pressure remained low. Arterial blood gas analysis showed pH of 7.4, PaCO2 of 42 mmHg, PaO2 of 272 mmHg (80-100 mmHg), and HCO3 of 26.3 mEq/L (22-28 mEq/L). His hemoglobin was 9.7 g/dL. Electrocardiogram (ECG) showed atrial fibrillation with rapid ventricular response at 120-140 beats per minute (Figure 1). The patient also received additional IV fentanyl to treat possible surgical pain.

To provide the best care to the patient, the attending physician and senior resident used the Stanford Anesthesia Emergency Manual checklist on the management of supraventricular tachycardia to guide the treatment. Multiple doses of 100 mcg bolus of phenylephrine were administered. The anesthesiologist then administered 20 mg of esmolol to temporarily control the tachycardia, with continued intermittent phenylephrine, which resulted in blood pressure stabilization with the mean arterial pressure (MAP) restored to greater than 65 mmHg. The patient was placed on an esmolol infusion at 50 μg/kg/min to maintain MAP for the remaining intraoperative time. During the operation, the patient was given total of 7,000 mL of IV normal saline. The patient was transferred to the post-anesthesia care unit with an endotracheal tube in place. His postoperative vital signs were significant for hypotension (102/63 mmHg) and tachycardia (138 bpm). Cardiology was consulted. The patient required continuous diltiazem for hemodynamic stability in the post-anesthesia care unit, where he was eventually transferred to the ICU. His remaining post-operative course was uneventful with no post-operative complications.

## Discussion

Intraoperative tachyarrhythmias are relatively common, but can lead to significant morbidity and mortality if not managed timely and appropriately [1-4]. Intraoperative supraventricular tachycardia with narrow QRS complexes can be treated with immediate synchronized cardioversion, adenosine, beta blockers, or nondihydropyridine calcium channel blockers, or some sequence or combination of these treatment modalities [12-13]. The entire clinical picture must be considered when deciding the best treatment modality for a patient because factors such as volume status, cardiac contractility, patient co-morbidities, and the feasibility of certain modalities often alter management.

Under normal circumstances, the anesthesiologist can use knowledge and experience to guide the management of uncomplicated intraoperative tachyarrhythmias. However, in the presented case, the patient developed an episode of supraventricular tachycardia and hypotension which was refractory to IV fluids and phenylephrine alone. Fortunately, the anesthesiologist decided to reference an EM before continuing with further management, rather than relying on rote memory. Although the initial decision of rate control with pharmacologic therapy versus immediate synchronized cardioversion typically depends on hemodynamic instability, electrical cardioversion would have mechanically interfered with critical steps of the exploratory laparotomy and was to be avoided in this circumstance unless deemed absolutely necessary [12-13]. The decision of which pharmacologic agent to use for rate control was ambiguous, as all three first-line agents can potentially lower blood pressure through different mechanisms. The Stanford Anesthesia EM allowed rapid identification of the most appropriate treatment option for this specific clinical scenario, which was esmolol [13]. The use of esmolol ultimately resolved the patient’s tachyarrhythmia and stabilized his blood pressure to a MAP > 65 mmHg.

The EMs are cognitive aids that help reduce medical error by providing immediate access to current, medically established guidelines on the management of intraoperative complications [8]. In our institution, each anesthesia station has one hard copy of the EM and most trainees have downloaded the digital copy on their smartphone for immediate use. Multiple simulation trainings of EM use have been conducted. EMs have been shown to improve the clinical outcome of critical events and reduce the incidence of missing key steps during crisis management [6, 14]. EMs also increase teamwork, efficiency, collaboration, and confidence [9-11, 14-16]. In the patient’s case, the use of an EM directly improved patient care and avoided any adverse event that may have occurred with selection of a different pharmacologic agent.

## Conclusions

The effective application of an EM to the management of intraoperative supraventricular tachycardia with refractory hypotension resulted in hemodynamic stabilization with no further complications. This case adds to the growing body of evidence that EMs should be readily available for utilization in critical events, as they improve patient care by providing immediate access to guideline-based resources and thus decreasing reliance on rote memory.

## References

[1] Amar D (2002). Perioperative atrial tachyarrhythmias. Anesthesiology.

[2] Melduni RM, Koshino Y, Shen WK (2012). Management of arrhythmias in the perioperative setting. Clin Geriatr Med.

[3] Reich DL, Bennett-Guerrero E, Bodian CA, Hossain S, Winfree W, Krol M (2002). Intraoperative tachycardia and hypertension are independently associated with adverse outcome in noncardiac surgery of long duration. Anesth Analg.

[4] Maisel WH, Rawn JD, Stevenson WG (2001). Atrial fibrillation after cardiac surgery. Ann Intern Med.

[5] Rothschild JM, Landrigan CP, Cronin JW (2005). The Critical Care Safety Study: the incidence and nature of adverse events and serious medical errors in intensive care. Crit Care Med.

[6] Hepner DL, Arriaga AF, Cooper JB, Goldhaber-Fiebert SN, Gaba D (2017). Operating room crisis checklists and emergency manuals. Anesthesiology.

[7] Leape LL (1994). Error in medicine. JAMA.

[8] Goldhaber-Fiebert SN, Howard SK (2013). Implementing emergency manuals: can cognitive aids help translate best practices for patient care during acute events. Anesth Analg.

[9] Goldhaber-Fiebert SN, Pollock J, Howard SK, Bereknyei Merrell S (2016). Emergency manual uses during actual critical events and changes in safety culture from the perspective of anesthesia residents: a pilot study. Anesth Analg.

[10] Huang J, Hoang P, Simmons W, Zhang J (2019). Free emergency manual books improve actual clinical use during crisis in China. Cureus.

[11] Huang J, Sanchez K, Wu J, Suprun A (2019). Best location and reader role in usage of emergency manuals during critical events: experienced emergency manual users’ opinions. Cureus.

[12] Stewart AM, Greaves K, Bromilow J (2015). Supraventricular tachyarrhythmias and their management in the perioperative period. Contin Educ Anaesth Crit Care Pain.

[13] Howard SK, Chu LF, Goldhaber-Fiebert SN, Gaba DM, Harrison TK (2020). Emergency Manual: Cognitive Aids for Perioperative Critical Events. Emergency Manual: Cognitive aids for perioperative critical events.

[14] Arriaga AF, Bader AM, Wong JM (2013). Simulation-based trial of surgical-crisis checklists. N Engl J Med.

[15] Simmons WR, Huang J (2019). Operating room emergency manuals improve patient safety: a systemic review. Cureus.

[16] Goldhaber-Fiebert SN, Lei V, Nandagopal K, Bereknyei S (2015). Emergency manual implementation: can brief simulation-based or staff trainings increase familiarity and planned clinical use?. Jt Comm J Qual Patient Saf.

